# APE1/Ref-1 – One Target with Multiple Indications: Emerging Aspects and New Directions

**Published:** 2021

**Authors:** Mahmut Mijit, Rachel Caston, Silpa Gampala, Melissa L. Fishel, Jill Fehrenbacher, Mark R. Kelley

**Affiliations:** 1 Herman B Wells Center for Pediatric Research, Department of Pediatrics, Indiana University School of Medicine, 1044 W. Walnut, Indianapolis, IN 46202, USA; 2 Department of Pediatrics, Indiana University School of Medicine, 1044 W. Walnut, Indianapolis, IN 46202, USA; 3 Department of Pharmacology and Toxicology, Indiana University School of Medicine, 1044 W. Walnut, Indianapolis, IN 46202, USA; 4 Indiana University Simon Comprehensive Cancer Center, Indiana University School of Medicine, 1044 W. Walnut, Indianapolis, IN 46202, USA; 5 Department of Biochemistry and Molecular Biology, Indiana University School of Medicine, 1044 W. Walnut, Indianapolis, IN 46202, USA

**Keywords:** Redox effector factor 1, Apurinic/apyrimidinic endonuclease, Redox signaling, Inflammation, Metabolism, Glycolysis, TCA cycle, OXPHOS, Chemotherapy-induced peripheral neuropathy, IBD, Crohn’s, Colitis

## Abstract

In the realm of DNA repair, base excision repair (BER) protein, APE1/Ref-1 (Apurinic/Apyrimidinic Endonuclease 1/Redox Effector - 1, also called APE1) has been studied for decades. However, over the past decade, APE1 has been established as a key player in reduction-oxidation (redox) signaling. In the review by Caston *et al.* (*The multifunctional APE1 DNA repair-redox signaling protein as a drug target in human disease),* multiple roles of APE1 in cancer and other diseases are summarized. In this Review, we aim to expand on the contributions of APE1 to various diseases and its effect on disease progression. In the scope of cancer, more recent roles for APE1 have been identified in cancer cell metabolism, as well as chemotherapy-induced peripheral neuropathy (CIPN) and inflammation. Outside of cancer, APE1 signaling may be a critical factor in inflammatory bowel disease (IBD) and is also an emergent area of investigation in retinal ocular diseases. The ability of APE1 to regulate multiple transcription factors (TFs) and therefore multiple pathways that have implications outside of cancer, makes it a particularly unique and enticing target. We discuss APE1 redox inhibitors as a means of studying and potentially combating these diseases. Lastly, we examine the role of APE1 in RNA metabolism. Overall, this article builds on our previous review to elaborate on the roles and conceivable regulation of important pathways by APE1 in multiple diseases.

## Introduction

APE1/Ref-1 (also called APE1) has been extensively studied, initially for its singular role as an endonuclease in DNA base excision repair (BER). However, since the early 2000’s, it has been studied for other roles that have been discovered in addition to DNA repair which include its major role as a reduction-oxidation (redox) signaling protein and its interactions with RNA [1]. Multiple studies report the many functions of APE1/Ref-1 in regulation of key biological functions that control redox homeostatsis [2–4], mitochondrial metabolism [5,6], inflammatory responses [7], neo-vascularization [8,9] and others that make it an attractive target in pathologies such as cancer, chemotherapy-induced peripheral neuropathy (CIPN), inflammatory bowel disease (IBD), retinal ocular diseases [e.g. diabetic retinopathy (DR), diabetic macular edema (DME), and wet age-related macular degeneration (Neovascular AMD)]. APE1’s functional roles are also associated with its localization either within the nucleus or cytoplasm (endoplasmic reticulum or mitochondria) [10,11]. Aditionally, secreted APE1 is reported to induce proinflammatory cytokine IL-6 [12]. APE1 serum levels serve as biomarkers for bladder cancer, hepatocellular carcinoma and oral squamous cell carcinoma [13–16].

APE1’s endonuclease role controlling DNA and RNA metabolism and its redox role regulating transcription factors are attractive targets and so far, several small molecule inhibitors have been developed and are successful in the pre-clinical setting. Only recently, APX3330, an APE1 redox function inhibitor completed Phase I clinical trial with a good safety profile, verified target engagement and a recommended phase II dose (600 mg/d). The review by Caston *et al.* (*The multifunctional APE1 DNA repair-redox signaling protein as a drug target in human disease),* focused mostly around APE1’s role in cancer and the use of inhibitors of the redox signaling function of APE1. In this article, we will address some of the less discussed or understudied roles of APE1 that are gaining more recent attention.

## APE1 and Cancer Cell Metabolism: New Discoveries

Cancer cells can adapt to meet their ever-changing bioenergetic needs by reprogramming their metabolic pathways thereby acquiring and maintaining unrestricted proliferative capability. Cancer cell metabolic adaptability might be possible due to accumulation of specific mitochondrial metabolites like fumarate, succinate and α-ketoglutarate [17]. To meet their constant demand for fuel especially under nutrient-deprived stress, it is likely that certain malignant cancers shift from glycolysis back to oxidative phosphorylation (OXPHOS) as the main energy supplier [18]. This bidirectional shift might be possible because of mitochondrial metabolic plasticity. In oncogene mutated cancers like KRAS^G12D^-driven PDAC (Pancreatic Ductal Adenocarcinoma) and MYC/KRAS or MYC/ERBB2-ablated breast cancer, cancer cells predominantly rely on OXPHOS for energy production [17]. Consequently, a previous notion that the Warburg effect is responsible for all tumor survival and growth proves inaccurate due to this dynamic interplay between oxidative metabolism and glycolysis.

**Figure 1:**
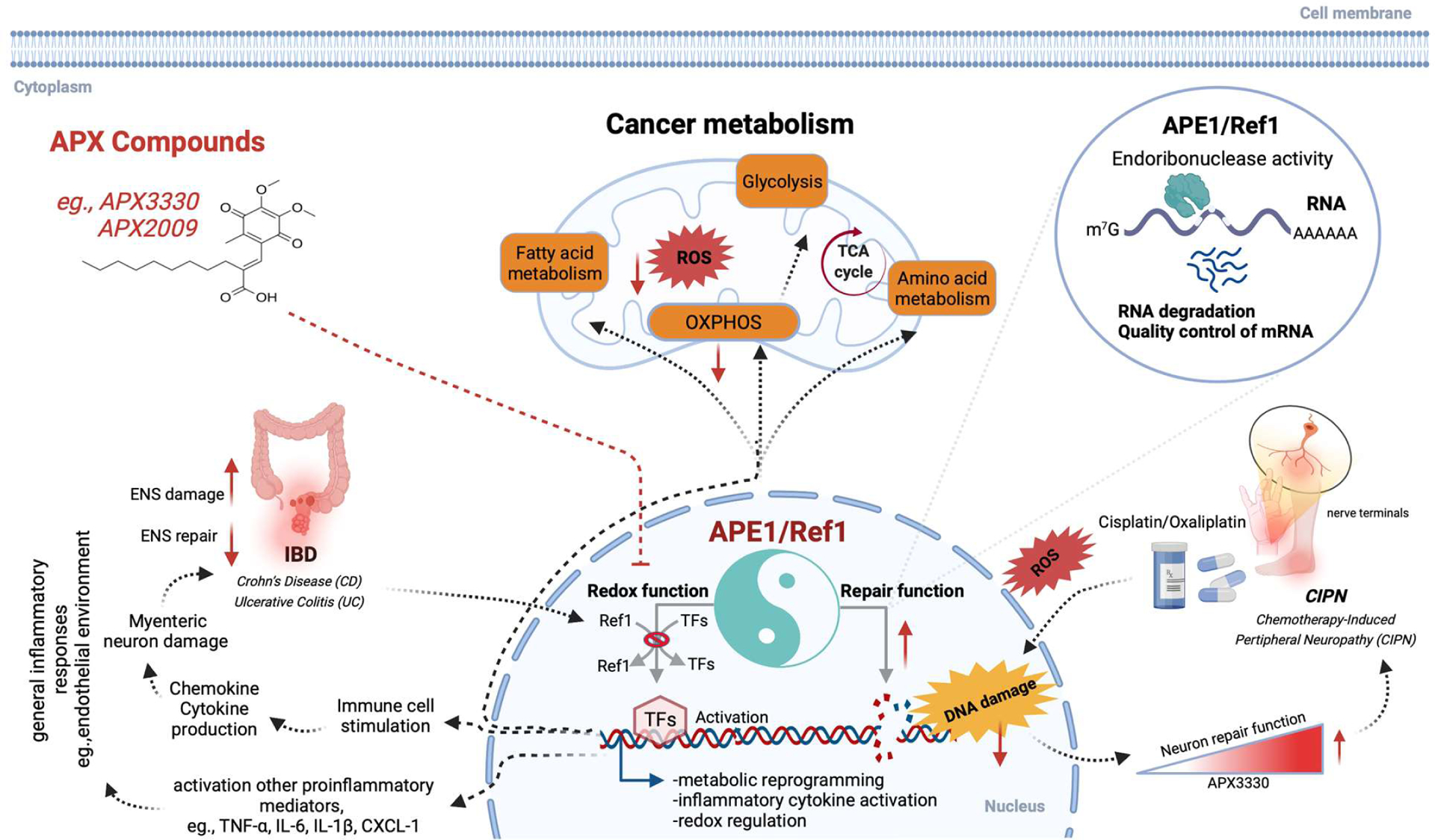
APE1/Ref-1 plays a vital role in DNA and RNA repair and reduction/oxidation (redox) signaling and impacts cancer cells metabolism and other inflammatory-driven pathologies. TF activation mediated by APE1 redox function is involved in cellular metabolism, angiogenesis as well as inflammation-derived human diseases such as IBD. Subsequent activation of downstream mediators (such as TNF-α, IL-1, etc.) also have direct impact on inflammatory responses. Inhibition of the redox signaling pathway with APX compounds reduces the overproduction of proinflammatory cytokines and chemokines and ROS production and alleviates inflammation. The repair function of APE1 is essential for repair of damaged DNA or RNA lesions caused by chemotherapy (e.g., Cisplatin or Oxaliplatin) or other environmental factors. Overall, APX compounds can reduce inflammatory markers and ROS levels, enhance DNA repair function especially in neurons, thereby providing a new therapeutic opportunity for wide range of human illness. **Abbreviations**: TFs: Transcription Factors; IBD: Inflammatory Bowel Disease; CIPN: Chemotherapy Induced Peripheral Neuropathy; OXPHOS: Oxidative Phosphorylation; ENS: Enteric Nervous System; TNF-α: Tumor Necrosis Factor alpha; IL-1/6: Interleukin (1/6); ROS: Reactive Oxygen Species; STAT3: Signal Transducer and Activator of Transcription 3; NFκB: Nuclear Factor kappa-light chain enhancer of activated B cells; AP-1: Activator Protein 1; HIF1α: Hypoxia Inducible Factor 1 subunit alpha; redox: Reduction-oxidation; KRAS^G12D^: Kirsten RAt Sarcoma virus; PDAC: Pancreatic Ductal Adenocarcinoma; MYC: MYeloCytomatosis protooncogene; ERBB2: ERythroBlastic oncogene B; EIF 2: Eukaryotic Initiation Factor 2; mTOR: Mammalian Target of Rapamycin; CD: Crohn’s disease; UC: Ulcerative Colitis; GI: Gastrointestinal; ENS: Enteric Nervous System; MNs: Myenteric Neurons; NLRP: NLR family pyrin domain containing proteins; NO: Nitric Oxide; PGE2: Prostaglandin E2; LPS: Lipopolysaccharide

The redox signaling activity of APE1 is responsible for facilitating the DNA binding of critical TFs like STAT3 (Signal transducer and activator of transcription 3), NFκB (Nuclear Factor kappa-light chain enhancer of activated B cells), AP-1 (Activator Protein 1) and HIF1α (Hypoxia inducible factor 1 subunit alpha) among others (Figure 1) [19]. APE1 reduces HIF1α thereby activating its DNA binding capability [20,21] further regulating HIF1α-dependent cancer metabolic alterations [22]. In our recently published review, we described a role for the redox activity of APE1 to regulate mitochondrial metabolism, in addition to its role in DNA repair and tumor cell survival and growth. Inhibition of APE1 endonuclease activity is reported to impair mitochondrial respiration in a p53-dependent manner [23]. APE1 when methylated at R301 (Arginine 301) [24] translocates to the mitochondria [25] through the mitochondrial intermembrane space import and assembly protein 40 (MIA) pathway [26] and is responsible for maintaining mitochondrial DNA (mtDNA) integrity under oxidative stress and drives mitochondrial respiration [25]. Other studies have shown that mitochondrial APE1 decreases reactive oxygen species (ROS) generation in osteosarcoma cancer cells thereby promoting cisplatin resistance [27]. Endogenous ROS is a constant threat to mtDNA and reports show that both truncated and full-length APE1 have been found to be capable of repairing mtDNA through its endonuclease activity [28–30].

Previously published single cell (sc) RNA-seq analysis of low passage pancreatic cancer patient derived cells (Pa03C) transfected with scrambled or siAPE-1 (small interfering RNA against APE1) revealed novel pathways downregulated with APE1 knockdown including EIF2 as well as mTOR signaling [19]. Under hypoxia, a cluster within scrambled control cells was found to have high expression of HIF1α-regulated genes, whereas that upregulation was abrogated by siAPE1 knockdown. Analysis of the same data identified glycolysis, Tricarboxylic acid (TCA) cycle, and OXPHOS pathways to be downregulated with APE1 knockdown especially under hypoxic conditions (Figure 1) [5].

At the time of publication of the review [1] all of our compiled data as well as the existing literature pointed toward APE1’s repair function within the mitochondria to be predominantly responsible for the observed changes in mitochondrial function. However, more recent data implicate APE1’s redox regulation of transcription factors including HIF1α using APX2009 (a potent second generation APE1 redox inhibitor derived from APX3330) to significantly affect mitochondrial metabolism. Subsequent results from our work revealed that APE1 redox activity strongly influences OXPHOS signaling [6].

All of these findings raise the question: how and what exactly is APE1 orchestrating to control mitochondrial metabolism that regulates and helps provide energy for cancer growth and metastasis? Do these findings suggest that APE1 redox inhibitors alone or in combination with other metabolism altering drugs could provide better treatment options in cancer? This is an area of intense study in our laboratories focusing on the underlying mechanisms of APE1 redox regulation of cancer cells and their mitochondrial metabolic plasticity.

In the Caston *et al.* review, we discussed the development of the APE1 redox inhibitor APX3330 and second-generation inhibitors APX2009 and APX2014 and others [1]. These compounds specifically inhibit the redox function of APE1; they do not inhibit the DNA repair activity of APE1. In fact, our data suggest that the compounds enhance the endonuclease repair activity of APE1 in sensory and enteric neurons [31,32]. Several publications support the notion that APX3330 and APX2009 are neuroprotective in preclinical disease models and that APE1 plays a role in not just in the tumor cells, but also in CIPN, a common side effect of cancer treatment [31,33–35].

## APE1 and Chemotherapy-Induced Peripheral Neuropathy (CIPN)

CIPN is a potentially debilitating side effect of a number of chemotherapy drugs and can lead to a reduction in the dose of chemotherapy thereby potentially limiting efficacy. This neuropathy is characterized by alterations in peripheral sensory function originating in the hands and feet, such as numbness and tingling, increased sensitivity to mechanical touch and cold, loss of proprioception, and reduced tendon reflexes depending on the involvement of sensory and motor nerves. Pain is a debilitating symptom in a subset of patients, who can experience stabbing, burning, and electrical sensations [36]. Treatment and preventative or therapeutic options are limited [37–39].

Previous work strongly supports the hypothesis that DNA damage in neurons is a major contributor to CIPN, especially for platinum-containing drugs (cisplatin, oxaliplatin, and carboplatin). These drugs induce platinum:DNA crosslinks and this adduct formation has been correlated with neuronal toxicity [40,41]. In addition to adduct formation, several platinum drugs also increase the formation of reactive oxygen species (ROS), which leads to oxidative DNA damage and neurotoxicity [19,42–47]. The repair of both endogenous and exogenous DNA damage is critical to maintain neuronal homeostasis [48–51]. Neurons contain a host of DNA repair pathways, including nucleotide excision repair (NER), which is responsible for removing bulky adducts, such as the platinum:DNA crosslinks and the BER pathway, which removes nonbulky damage such as base oxidation and alkylation within nuclear and mitochondrial DNA. Because neurons are highly metabolic [52], there is a steady production of ROS and subsequent oxidative DNA damage, thus the BER pathway predominates as the primary DNA repair pathway in neurons [50,53,54]. APE1 is a key player in BER, hydrolyzing the phosphodiester backbone immediately 5′ to an apurinic/apyrimidinic (AP) site, generating a normal 3′-hydroxyl group and an abasic deoxyribose-5-phosphate, which is processed by subsequent enzymes of the BER pathway. As mentioned above, APE1 is targeted to the mitochondria and is responsible for the repair of both nuclear and mitochondrial DNA. Compromising the repair activity of APE1 would allow oxidative DNA damage to accumulate in neurons, whereas enhancing APE1 repair activity could reduce damage [31,33,34,50].

Our previously published work has demonstrated that overexpression of APE1 protects sensory neurons from oxidative DNA damage, whereas reduction of APE1 expression leads to increased cytotoxicity after neurons were exposed to oxidative DNA damaging agents or chemotherapeutic agents that are known to generate ROS and subsequent oxidative DNA damage [31,33,34,50]. The neuronal protection provided by overexpressing APE1 is recapitulated by APX3330 and APX2009 [33,55–57]. In rat dorsal hind paw skin, sensory nerve-induced vasodilation can be used to assess the function of sensory neurons that innervate the dermis. Systemic administration of cisplatin decreases acute vasodilation induced by sensory nerve activation, whereas treatment of the rats with APX3330 partially restored the vasodilation, suggesting that APX3330 was neuroprotective [31]. Furthermore, the APX compounds do not decrease the efficacy of chemotherapeutics in cancer cell lines or in tumor-bearing mice [56]. The ability of the small molecule APX compounds to enhance APE1-mediated DNA repair activity to diminish the neurotoxic effects of chemotherapeutics without compromising antitumor effects provides a novel means to prevent or reverse CIPN [31,34,35].

## APE1 in Inflammatory Bowel Disease (IBD)

IBD is characterized by pathological epithelial injury in two main types of intestinal inflammatory conditions: Crohn’s disease (CD) and ulcerative colitis (UC) [58]. In 2015, approximately 1.3% of US adults (3 million) reported being diagnosed with either CD or UC [59]. The UC inflammation is usually induced in the mucosal layer, and typically characterized by chronic bloody diarrhea, tenesmus, and abdominal pain. However, CD describes transmural (full-thickness) inflammation, affecting all layers of the gut wall, can be extended to any part of the gastrointestinal tract (GI) mostly terminal ileum and colon [60]. A long-term complication of IBD is the development of colorectal cancer [58].

GI tract immune dysregulation is a key factor of IBD onset as it is accompanied by a considerable infiltration of inflammatory cells in the gut mucosa [61]. Activation of immune cells and the release of ROS are prominent early events in the pathogenesis of IBD that could dramatically affect the Enteric Nervous System (ENS) [62]. Enteric glial cells are known to actively be involved in inflammatory processes as they can produce and respond to proinflammatory cytokines (e.g., IL-1β, TNF-α, IL-6, etc.) [63]. Myenteric neurons (MNs) are main constituents of ENS located between the longitudinal and circular layers of muscularis externa in the gastrointestinal tract. MNs are also known as major coat of the intestinal wall, mainly responsible for functioning bowel movement. Inflammation and oxidative stress have profound impact on myenteric plexus that could also induce neuronal loss in the colon [64]. For example, in UC rat model, the numbers of MNs were reduced nearly by 50% in comparison to control group [65]. Similar observation was found in a clinial study, there was a significant decrease in the number of MNs (from 604.89 ± 29.65/mm^2^ to 237.56 ± 59.93/mm^2^) in patients with UC [66], highlighting the fact that neurotoxic insult on myenteric plexus is a principal cause of IBD. Therefore, the DNA repair and removal mechanisms that cope with oxidative stress-mediated DNA damage must also be considered in addition to the proinflammatory cytokine profile when treating IBD.

Several preclinical investigations demonstrate that APE1 is an effective target in treating IBD [32,67]. For instance, Chang *et al.* induced IBD in rats with dextran sulfate sodium (DSS) administration, which initially induces colonic injury, and then further causes chronic colitis over the time. They revealed that APE1 and MSH2 (mismatch repair gene) levels were significantly increased in a time-dependent manner with DSS-treatment [68]. In addition, DNA damage marker 8-hydroxy-deoxyguanosine (8-OHdG) was increased in the colonic mucosa, whereas APE1 levels in the surface epithelium increased at an earlier timepoint, highlighting the fact that APE1 is a sensitive target for determining exacerbation of DNA damage in DSS-induced colitis [68]. Thus, APE1 may control or contribute to earlier cellular/molecular events in pathogenesis of DSS-induced colitis.

More recent studies by Sahakian *et al.* established a colitis model in mice that develop defective epithelium which resembles the pathophysiology of human IBD [69]. They demonstrated increased levels of APE1 in both the mucosa and myenteric ganglia as well as overproduction of ROS in mitochondria that caused oxidative DNA damage and translocation of the DNA damage marker HMGB1 from nuclei to cytoplasm in the mouse colons [69]. Furthermore, targeting APE1 redox activity with APX3330 lessened disease severity, reduced immune cell infiltration, restored GI function, and thereby provided neuroprotective effects to the ENS [69]. Most importantly, the repair activity of APE1 was enhanced while APX3330 inhibited the redox activity, suggesting that increases in redox activity and DNA damage both underlie the pathological bowel inflammation and provides a potential therapeutic approach for treating IBD. This finding of APE1 redox inhibition by APX3330 with enhancement of APE1 repair function is similar to what was seen in dorsal root ganglion (DRG) neurons in the CIPN models [31,33–35].

## APE1/Ref-1 in Inflammation-Associated Diseases

Inflammation is a biological response of the immune system that is induced by various invading pathogens and/or endogenous stimuli and essential for tissue healing processes [70]. Chronic inflammation, however, disrupts cellular homeostasis and leads to diseases, such as cardiovascular and bowel diseases, diabetes, osteoarthritis, and various cancers [71].

Cytokines are cell signaling proteins produced primarily by immune cells, including macrophages, dendritic cells, lymphocytes, tumors, and cancer associated fibroblasts in response to different stimulation [71,72]. However, certain cytokines, such as tumor necrosis factor-α (TNF-α), and interleukin-6 (IL-6), can be also induced by tumor cells as well as non-cancerous cell types, such as epithelial cells or cancer-associated fibroblasts (CAFs) [73]. A “cytokine storm” is an event that describes activation of an aggressive inflammatory response with the release of a large amount of pro-inflammatory cytokines, such as IL-1, TNF-α, and IL-6 [74]. The release of these cytokines is a major driver in most inflammatory disorders, including Severe Acute Respiratory Syndrome Coronavirus 2 (SARS-CoV-2) (COVID-19) [75]. Increasing numbers of clinical studies support that cytokine dynamics directly correlate to the clinical severity of COVID-19 infections, suggesting a central role of pro-inflammatory cytokines in respiratory diseases and activation of the adaptive immune response [76]. Cytokine receptor activation triggers important intracellular signaling pathways, including the mitogen-activated protein kinase (MAPK), nuclear factor kappa-B (NF-κB), and Janus kinase (JAK)-signal transducer and activator of transcription (STAT). For instance, more than 50 cytokines (such as IL-6 and others) found to be regulated via JAK/STAT signaling cascade induces inflammation and control the immune response [77]. Similarly, several other cytokines (such as TNF-α, IL-1 and IL-17) signaling cascades partially overlap and utilize some of the same mediators leading to the activation of some common TFs, such as NF-κB, AP-1, STAT3, ultimately leading to the regulation of gene expression [78].

As previously mentioned, APE1 is a redox catalyst involved with various biological processes by virtue of reducing several important TFs to enhance their binding to DNA, including STAT3, NF-κB, AP-1 and others involved in inflammatory regulation and response [19,30,79]. APE1 mediation of these key TFs and their activation of cytokines, chemokines, and other downstream targets promote growth, migration, and survival in tumor cells as well as angiogenesis in the tumor microenvironment [80]. A bioinformatics analysis demonstrated potential AP-1 and NF-κB binding sites within the promoter sequences of NLR family pyrin domain containing proteins (NLRP), namely *NLRP1* and *NLRP3*, which are key biomarkers of multiprotein intracellular complexes known as inflammasomes [81]. Indeed, Tang *et al.* demonstrated that APE1 promoted lipopolysaccharide (LPS)-induced NLRP3 inflammasome activation, along with overproduction of pro-inflammatory cytokines such as TNF-α, IL-1β, and IL-18 in tumor-associated macrophages through an NFκB-dependent pathway. In the same study, they showed that transcriptional levels of *TNF-α* and *NLRP3* along with other inflammasomes-associated molecules *Caspase-1* and *ASC* were significantly inhibited by both APX3330 and APE1 protein knockdown in THP-1 cells, suggesting the involvement of an APE1-NLRP3 axis in inflammasome pathways [82]. Jedinak *et al.* demonstrated that APX3330 suppressed secretion of inflammatory cytokines, including TNF-α, IL-6, IL-12 and the inflammatory mediator nitric oxide (NO) as well as prostaglandin E2 (PGE2) in LPS-stimulated macrophages through inhibition of TFs NF-κB and AP-1, both well-known primary targets of APE1 [83]. Taken together, these studies underscore the regulatory role of APE1 in various inflammatory processes by controlling important TFs that mediate proinflammatory cytokines.

## APE1 Role in RNA Metabolism

RNA is susceptible to base oxidation and alkylation, but how this damage is resolved is not fully understood. Some damage may be repaired via the protein hABH3RNA. However, some processing will likely occur through RNA metabolism to remove mutated RNA strands and damaged nucleotides from the RNA pool. Damaged RNA can lead to defective or mutant proteins during translation and likely inhibit reverse transcriptase activity, potentially contributing to cancer, schizophrenia, or muscle dystrophy [84,85].

APE1 has been characterized as a regulator of mRNA, which removes lesions from oxidative RNA substrates [86]. When APE1 is knocked down in HeLa cells, the number of oxidized mRNA increased, implying that APE1 is involved in mRNA cleansing [86]. APE1 is also capable of cleaving mRNA using its endonuclease activity, causing RNA degradation [86–90]. Currently, APE1 is known to cleave cMyc, SARS and CD44 [90].

APE1 also enhances post-translational maturation of microRNA. Specifically, APE1 has been shown to interact with the DROSHA complex to cleave microRNA-221/222, an oncogenic miRNA when upregulated, causing thyroid papillary carcinomas, glioblastomas, prostate carcinoma, and gastric carcinoma [91]. Binding of APE1 to DROSHA was increased under conditions of oxidative stress. Increased cleavage of pre-miRNA-221/222 by APE1 led to cancer cell growth *in vitro*. A similar result was seen with miR-92b, which enhances cervical carcinoma [92]. Therefore, dysregulation of APE1’s endonuclease function further supports the evidence that APE1 plays a role in not only DNA repair, but RNA metabolism as a cleansing mechanism of damaged RNA, with implications for cancer progression.

## Summary and Conclusion

In this review, we have highlighted the recent advances in our understanding of APE1 signaling in various pathological conditions, namely CIPN, IBD, as well as general inflammatory signaling along with possible pharmacological interventions (Figure 1). Initially, we discussed how APE1’s redox function alters mitochondrial metabolism during cancer progression. APE1 is present in both the nucleus and the mitochondria; its impact on metabolism could stem from its role in DNA repair as well as its role in regulating the activity of various TFs involved in gene regulation.

Additionally, we focused on APE1 in neuronal pathologies, we have discussed that selective inhibition of APE1’s redox function, without impeding the repair function, leads to increased DNA repair activity of APE1. In CIPN, the neurotoxic effects of platinating agents were diminished without compromising their anti-tumor properties through inhibition of APE1’s redox signaling. Similarly, in IBD, inhibiting APE1’s redox signaling had a neuroprotective effect, as well as anti-inflammatory, lessening the disease severity and enhancing APE1 DNA repair capacity, specifically with a reduction in oxidative DNA damage.

Finally, we explored APE1’s role in microRNA maturation and therein disease progression. Unlike CIPN and IBD, which have been linked to mis-regulated redox signaling, APE1’s interaction with microRNAs is dependent on its endonuclease activity.

In conclusion, APE1’s redox and DNA repair capabilities are interwoven in the cellular response to cancer metabolism, neuropathy, inflammation, and RNA processing. APE1 is a “hotspot” for inflammatory-oxidative connections, which provide a new therapeutic opportunity for treating a wide range of debilitating human diseases.
